# Self-efficacy in lifestyle management among young Arab adults: a cross-cultural study in the EMRO region

**DOI:** 10.3389/fpubh.2026.1633934

**Published:** 2026-02-05

**Authors:** Abeer Salman Alzaben, Samira Mahboub, Nahla Mohammed Bawazeer, Fatmah Almoayad, Huny M. Bakry, Howeida H. Abusalih, Yasir Najah Hussein, Laila Alkilani, Nada Benajiba

**Affiliations:** 1 Department of Health Sciences, College of Health and Rehabilitation Sciences, Princess Nourah bint Abdulrahman University, Riyadh, Saudi Arabia; 2 Collage of Physical Education and Sport Sciences, University of Baghdad, Baghdad, Iraq; 3 Department of Physical Sport Sciences, College of Sport Sciences & Physical Activity, Princess Nourah bint Abdulrahman University, Riyadh, Saudi Arabia; 4 Unité Mixte de Recherche en Nutrition et Alimentation URAC 39 (Université Ibn Tofaïl–CNESTEN), RDC-Nutrition, Kénitra, Morocco

**Keywords:** Arabian Peninsula, eastern Mediterranean, knowledge, northern Africa, nutrition, self-efficacy

## Abstract

**Objectives:**

The aim of this study was to assess the self-efficacy of lifestyle practices among young adults, including late adolescents aged 18–19 years, across the Arab region.

**Methods:**

A multinational cross-sectional online survey was conducted using convenience sampling between September 2022 and September 2023 (*n* = 2,708) across multiple Arab regions (the Arabian Peninsula, Africa, and the Eastern Mediterranean, but outside African countries). Individuals of both sexes, aged 18–25 years, who could read and understand Arabic and who resided in one of the Arab regions, were included. Sociodemographic data were also collected. A previously validated Arabic questionnaire was used to assess self-efficacy and knowledge of healthy lifestyles.

**Results:**

Individuals in the Northern Africa region (9.7 ± 3.5) had significantly lower total self-efficacy scores than those in the Arabian Peninsula (11.5 ± 2.8) and Eastern Mediterranean region (11.7 ± 2.7; *p* < 0.001, *F* = 132.3). The knowledge score was significantly lower in Northern Africa (5.4 ± 2.3), whereas the Arabian Peninsula had the highest knowledge score (7.2 ± 1.9; *p* < 0.001, *F* = 173.2). Knowledge was associated with self-efficacy in the Arabian Peninsula (R^2^ = 0.013, *p* = 0.016). Age and sex were related to self-efficacy in the Eastern Mediterranean region (R^2^ = 0.01, *p* = 0.007). In North Africa, age, sex, and knowledge were related to self-efficacy (R^2^ = 0.25, *p* < 0.001). These findings indicate that regional differences are related to cultural factors such as age, sex, and knowledge.

**Conclusion:**

Self-efficacy was lower in Northern African countries than in the Arabian Peninsula or Eastern Mediterranean countries. Age, sex, and nutritional knowledge were associated with self-efficacy. These findings may guide culturally sensitive public health interventions in Arab countries. Future research should explore these relationships using longitudinal designs to better understand the causal pathways between culture, knowledge, sociodemographic factors, and self-efficacy.

## 1 Introduction

Self-efficacy, defined as the belief of an individual in their capability to execute practices necessary to produce specific performance attainments (1, 2). In the context of young adults and adolescent health, self-efficacy has been increasingly examined for its role in shaping dietary habits, physical activity, and overall health outcomes (2, 3). Evidence has indicated that higher self-efficacy enhances the capacity to adopt and maintain healthy lifestyle habits and is associated with improved anthropometric outcomes, such as reduced abdominal obesity indices (2, 4). Steele et al. reported similar findings where specific self-efficacy management was related to healthy eating and physical activity, which can predict adiposity indicators (3). These studies support the concept that self-efficacy is a predictor of dietary practice change as well as an influential factor in physical health outcomes among teenagers.

Research has shown that several factors influence Arab young adults and adolescent self-efficacy in adopting healthier lifestyles. Bani-Issa et al. reported that parental and school support can enhance diet and exercise self-efficacy (5). Musaiger et al. identified a lack of information, motivation, and time as major barriers to healthy eating and physical activity (6). In a recent study, AlGhanim and Alkazemi examined these factors in Kuwait and determined that, although adolescents reported high levels of self-efficacy, targeted interventions were still required to translate these beliefs into sustained healthy behaviors (7). Collectively, these studies stress the importance of addressing sociocultural barriers and supporting self-efficacy among adolescents and young adults in Arab countries.

Although the study population spans ages 18–25 years, which is commonly classified as young adulthood, individuals aged 18–19 years may also be conceptualized as late adolescents according to developmental frameworks (8). In this study, participants are therefore analyzed within a young adult framework, while recognizing late adolescence as a transitional developmental phase. This approach allows conceptual continuity with the adolescent self-efficacy literature while maintaining definitional coherence.

Arab countries are culturally and historically rich with extensive ethnic, social, and economic diversity that may influence self-efficacy and health-related behaviors (9–11). Despite this heterogeneity, few studies have examined self-efficacy in Arab populations, even though many countries share overarching linguistic, religious, and sociocultural characteristic. To facilitate meaningful cross-national comparisons while accounting for contextual differences, Arab countries were grouped into three sub-regions: the Arabian Peninsula, North Africa, and Eastern Mediterranean, a classification commonly used in regional health, demographic, and epidemiological research (10). This approach allows for the examination of broad regional patterns while acknowledging underlying sociopolitical and health system differences.

The Arabian Peninsula (Saudi Arabia, Kuwait, the United Arab Emirates, Yemen, Bahrain, Oman, and Qatar) largely consists of high-income countries with well-developed healthcare systems and growing investment in preventive and digital health initiatives (12, 13); however, prevailing social norms may still shape lifestyle behaviors and levels of health-related autonomy among young adults (5). North African countries (Morocco, Algeria, Libya, Egypt, and Sudan) are mostly middle-income nations with developing health infrastructure and significant disparities in access to healthcare services and health education, particularly between urban and rural areas (14). The Eastern Mediterranean (Syria, Iraq, Lebanon, and Jordan) faces distinct public health challenges because of large refugee populations, which place additional pressure on health systems and may affect health behaviors and self-efficacy (15, 16). However, this region benefits from strong community engagement and active civil society organizations that promote community-based health initiatives, particularly in urban areas (15, 17).

Grouping countries into these three culturally and epidemiologically distinct sub-regions provides a more meaningful framework for comparing self-efficacy and health practices among young Arab adults. Accordingly, this study examined the differences in self-efficacy among young adults aged 18–25 years old across Arab countries and explored factors contributing to regional variation.

Despite growing interest in self-efficacy and lifestyle behaviors in Arab contexts, existing studies remain largely country-specific and methodologically heterogeneous. While several studies report positive associations between self-efficacy and healthy practices, others note high self-efficacy without sustained behavioral change, suggesting the influence of unmeasured structural and environmental barriers. Furthermore, the relative contribution of age, sex, and nutritional knowledge appears inconsistent across studies, limiting generalizability. Importantly, few studies have examined these relationships across multiple Arab regions using a unified methodological framework. This gap highlights the need for cross-regional analyses that account for cultural and sociodemographic variation, which the present study seeks to address.

## 2 Methods

### 2.1 Study design and participants

This cross-sectional study was conducted between September 2022 and September 2023 among young Arab adults using convenience sampling. The research protocol was approved by the Institutional Review Board of Princess Nourah Bint Abdulrahman University (number: 22–0406; date: Aug 24, 2022). An online self-administered survey was used, and participants who chose to complete the survey provided their consent by clicking on the “I agree” button. Individuals of both sexes aged 18–25 years, Arab citizens who could read and understand Arabic, and those who resided in an Arabic-speaking country at the time of the study were included. Any health conditions that could affect the dietary intake and choices of participants, such as diabetes, hypertension, cardiovascular or renal diseases, or adherence to a special diet, were excluded from the study.

### 2.2 Sampling and data collection

The study used a convenience-based snowball sampling approach. The online survey was disseminated through social media platforms (e.g., WhatsApp), and participants were encouraged to share the survey link within their networks. While this approach facilitated recruitment across multiple Arab countries, it may have introduced selection bias, including overrepresentation of individuals with internet access, higher digital literacy, or greater interest in health-related topics.

### 2.3 Study instruments

#### 2.3.1 Sociodemographic information

The survey collected sociodemographic information, including age (< 21 and 21–25 years), sex (male or female), marital status (single, married, widowed, or divorced), educational level (college degree or less than college degree), and current country of residence.

#### 2.3.2 The nutritional knowledge and self-efficacy questionnaire

The second part of the survey consisted of a previously translated Arabic nutritional knowledge and self-efficacy questionnaire administered to young Arabs (18). Its validity and reliability were assessed through a pilot study involving 50 Arabic-speaking adolescents and young adults from 22 Arab countries, with Cronbach’s alpha coefficients ranging from 0.59–0.76, indicating acceptable internal consistency (18).

#### 2.3.3 Nutritional knowledge

Nutritional knowledge was assessed using 10 questions, each with one correct answer. Participants received 1 point for each correct response, with a maximum possible score of 10 points. A score of < 50% of the maximum total score (≤ 4 points) was classified as poor knowledge, a score of 50–75% of the maximum (5–6 points) was considered fair knowledge, and a score of 75% or higher (7 points or more) was considered good knowledge.

#### 2.3.4 Self-efficacy

Overall, self-efficacy in lifestyle management was assessed using seven questions. The scores for each self-efficacy question were as follows: no = 0, I am not sure = 1, and yes = 2. The maximum possible score was 14 points. Overall self-efficacy in lifestyle management was assessed by self-efficacy in diet management (four questions, maximum score 8), practicing exercise (two questions, maximum score 4), and weight management (one question, maximum score 2). The overall self-efficacy score was calculated as the sum of all self-efficacy questions.

### 2.4 Statistical analysis

For analytical purposes, participating countries were grouped into three regions based on commonly used regional classifications in Arab and Middle Eastern health research: the Arabian Peninsula (Saudi Arabia, Kuwait, United Arab Emirates, Yemen, Bahrain, Oman, Qatar), North Africa (Morocco, Algeria, Libya, Egypt, Sudan), and the Eastern Mediterranean region outside Africa (Iraq, Syria, Lebanon, Jordan). Data were analyzed using JMP software version 14.2. Categorical variables are presented as frequency tables. One-way analysis of variance (ANOVA) and the post-hoc Scheffé test were conducted to compare self-efficacy and knowledge scores between regions. Cohen’s d test was used to measure the effect size between the study groups. A two-way ANOVA was performed to investigate the cultural effects of different regions in Arab countries on self-efficacy in changing lifestyles across different knowledge levels. A multiple linear regression model was used to assess the association between knowledge and sociodemographic characteristics and self-efficacy regarding lifestyle changes in each region. Statistical significance was set at *p* < 0.05.

## 3 Results

### 3.1 Sociodemographic

The sociodemographic distribution of the study sample across different regions of Arab countries is presented in Table 1. The Arabian Peninsula had a higher percentage of females than males, and most of the participants were single. The Eastern Mediterranean region has an equal percentage of males and females. Northern Africa had an equal percentage of males and females with a college degree (94%), whereas around 6% had a high school education or less.

**Table 1:** Sociodemographic distribution of the studied sample across different regions in the Arab world (*n* = 2,708).

Sociodemographic	Different regions in the Arab world					
	Arabian Peninsula (*n* = 627)		Eastern Mediterranean region (*n* = 1,012)		Northern Africa (*n* = 1,069)	
	N	(%)	N	(%)	N	(%)
Age						
less than 21 years	241	(38.44)	317	(31.32)	393	(36.76)
21–25 years	386	(61.56)	695	(68.68)	676	(63.24)
Gender						
Female	536	(85.49)	598	(59.09)	533	(49.86)
Male	91	(14.51)	414	(40.91)	536	(50.14)
Marital status						
Single	517	(82.46)	819	(80.93)	1,011	(94.57)
Married	104	(16.59)	172	(17.0)	45	(4.21)
widow or divorced	6	(0.96)	21	(2.08)	13	(1.22)
Level of education						
College degree	485	(77.35)	868	(85.77)	1,010	(94.48)
Less than college	142	(22.65)	144	(14.23)	59	(5.52)

N, number; %, percentage.

### 3.2 Self-efficacy and knowledge score

Table 2 shows the self-efficacy in lifestyle management and knowledge scores across different regions of Arab countries. Northern Africa had significantly lower scores in self-efficacy in changing weight and total self-efficacy score in managing lifestyle than the Arabian Peninsula and Eastern Mediterranean region (*p* < 0.001) with moderate effect size for all (Cohen’s d ˃0.5, *p* < 0.01). Self-efficacy in changing weight was significantly lower in Northern Africa than in the Arabian Peninsula and Eastern Mediterranean (Cohen’s d < 0.5, *p* < 0.01). Self-efficacy in practicing exercise was highest in the Eastern Mediterranean region and lowest in Northern Africa (*p* < 0.001) with a moderate effect size for all regions (Cohen’s d ˃0.5, *p* < 0.01). Knowledge was highest in the Arabian Peninsula and lowest in Northern Africa (Cohen’s d < 0.5, *p* < 0.001). These findings indicate that North African youth perceive lower self-efficacy and knowledge scores than their Arabian Peninsula and Eastern Mediterranean peers.

**Table 2:** Self-efficacy in lifestyle management and knowledge score across different regions in the Arab world (*n* = 2,708).

Self-efficacy	Arab Region						ANOVA *F* value	*p* value
	Arabian Peninsula (*n* = 627)		Eastern Mediterranean region (*n* = 1,012)		Northern Africa (*n* = 1,069)			
	M	(SD)	M	(SD)	M	(SD)		
Self-efficacy in changing diet^1^	6.7 ^a^	(1.7)	6.8 ^a^	(1.7)	5.7 ^b^	(2.1)	92.8	<0.001
Self-efficacy in practicing exercise^2^	3.2 ^a^	(1.2)	3.5 ^b^	(0.9)	2.7 ^c^	(1.3)	119.2	<0.001
Self-efficacy in changing weight^3^	1.6 ^a^	(0.7)	1.5 ^a^	(0.8)	1.3 ^b^	(0.8)	41.3	<0.001
Total self-efficacy score in managing lifestyle^1^	11.5 ^a^	(2.8)	11.7 ^a^	(2.7)	9.7 ^b^	(3.5)	132.3	<0.001
Total knowledge score^4^	7.2 ^a^	(1.9)	6.7 ^b^	(1.9)	5.4 ^c^	(2.3)	173.2	<0.001

M, mean; SD, standard deviation; *p*, significance level. ^a–c^Different superscripts indicate a significant difference *p* < 0.001.

1 Arabian Peninsula and Eastern Mediterranean region> Northern Africa, Cohen’s d ˃0.5.

2 Eastern Mediterranean region> Arabian Peninsula > Northern Africa, Cohen’s d ˃0.5.

3 Arabian Peninsula and Eastern Mediterranean region> Northern Africa, Cohen’s d < 0.5.

4 Arabian Peninsula >Eastern Mediterranean region > Northern Africa, Cohen’s d < 0.5.

### 3.3 Factors associated with self-efficacy in lifestyle change across different regions of the Arab world

Table 3 shows that the effect of culture on self-efficacy in changing lifestyles across different regions of Arab countries differs significantly across knowledge levels. In the Eastern Mediterranean region, the mean self-efficacy score is almost the same across different knowledge levels (11.8 ± 3, 11.8 ± 2.7, and 11.6 ± 2.6). However, in the Arabian Peninsula and Northern Africa region, the mean self-efficacy score is significantly lower at the poor knowledge level compared with that at good levels (10.6 ± 3.3 vs. 11.7 ± 27 in the Arabian Peninsula and 7.6 ± 3.4 vs. 11.6 ± 2·6 in Northern Africa; *F* = 34.2, *p* < 0.01). These findings indicate that nutritional knowledge is associated with self-efficacy.

**Table 3:** The effect of culture on self-efficacy in changing lifestyle across different levels of knowledge about healthy lifestyle.

Nutrition knowledge	Different regions in the Arab world								Two-way ANOVA F value	*p*-value
	Arabian Peninsula (*n* = 627)		Eastern Mediterranean region (*n* = 1,012)		Northern Africa (*n* = 1,069)		All			
	Total score of self-efficacy									
	M	(SD)	M	(SD)	M	(SD)	M	(SD)		
Poor knowledge	10.6	(3.3)	11.8	(3.0)	7.6	(3.4)	8.9	(3.7)	34.2	<0.010
Fair knowledge	11.4	(2.7)	11.8	(2.7)	10.5	(3.0)	11.2	(2.9)		
Good knowledge	11.7	(2.7)	11.6	(2.6)	11.6	(2.6)	11.6	(2.6)		
Average score	11.5	(2.8)	11.7	(2.7)	9.7	(3.5)	10.9	(3.2)		

M, mean; SD, standard deviation; *p*, significance level.

Table 4 shows the multiple linear regression model to measure the factors associated with self-efficacy in lifestyle change across different regions of the Arab regions. In the Arabian Peninsula, knowledge was significantly associated with self-efficacy (R^2^ = 0.013, *p* = 0.016). In the Eastern Mediterranean region, age and sex were significantly associated with self-efficacy (R^2^ = 0.01, *p* = 0.007). In North Africa, age, sex, and knowledge explained a larger proportion of the variance in self-efficacy (R^2^ = 0.25; *p* < 0.001).

**Table 4:** Factors associated with self-efficacy in lifestyle change across different regions of the Arab world (*n* = 2,708).

Model		Arabian Peninsula*							Eastern Mediterranean region**							Northern Africa***						
		Unstandardized coefficients		Standardized coefficients	t	*p* value	Collinearity statistics		Collinearity statistics		Standardized coefficients	t	*p* value	Collinearity statistics		Unstandardized coefficients		Standardized coefficients	t	*p* value	Collinearity statistics	
		B	SE	Beta			Tolerance	VIF	B	SE	Beta			Tolerance	VIF	B	SE	Beta			Tolerance	VIF
1	Constant	9.387	1.148		8.178	<0.001			9.758	0.879		11.107	<0.001			2.557	1.025		2.495	0.013		
	Age	0.028	0.046	0.025	0.619	0.536	0.99	1.01	0.073	0.037	0.063	1.988	0.047	0.98	1.01	0.262	0.045	0.155	5.783	<0.001	0.97	1.02
	Sex	0.317	0.318	0.040	0.999	0.318	0.98	1.02	0.373	0.175	0.068	2.131	0.033	0.96	1.04	−1.060	0.202	−0.153	−5.247	<0.001	0.83	1.20
	Knowledge	0.163	0.059	0.111	2.775	0.006	0.99	1.01	−0.023	0.044	−0.016	−0.512	0.609	0.97	1.03	0.570	0.044	0.376	12.826	<0.001	0.82	1.22
2	Constant	10.248	0.438		23.416	<0.001			9.603	0.825		11.643	<0.001									
	Age								0.073	0.037	0.062	1.969	0.049	0.98	1.01							
	Sex								0.388	0.173	0.071	2.247	0.025	0.98	1.01							
	Knowledge	0.167	0.059	0.113	2.850	0.005	1.00	1.00														

SE, Standard Error; p, significance level. *Model 1: R = 0.122, R^2^ = 0.015, *p* = 0.0025; Model 2: R = 0.113, R^2^ = 0.013, *p* = 0.016. **Model 1: R = 0.101, R^2^ = 0.010, *p* = 0.007; Model 2: R = 0.099, R^2^ = 0.010, *p* = 0.007. ***Model 1: R = 0.50, R^2^ = 0.25, *p* < 0.001.

## 4 Discussion

In this study, we examined cross-cultural differences in self-efficacy related to lifestyle management among young adults in Arab countries, focusing on the Arabian Peninsula, North Africa, and Eastern Mediterranean regions. Our findings reveal that cultural, demographic, and educational contexts play critical roles in shaping self-efficacy beliefs that are essential for sustaining healthy lifestyle practices in adolescents and young adults.

Participants from Northern Africa had the lowest self-efficacy and nutritional knowledge. In contrast, young adults from the Eastern Mediterranean region scored higher in self-efficacy in practicing exercise, and participants from the Arabian Peninsula scored the highest in nutritional knowledge compared to their peers. Moreover, the study revealed that knowledge was associated with self-efficacy scores in the Arabian Peninsula. Age and sex are associated with self-efficacy scores in the Eastern Mediterranean region. Age, sex, and nutrition knowledge were associated with self-efficacy scores in northern Africa.

### 4.1 The role of self-efficacy in lifestyle practices

Self-efficacy, defined as the belief of an individual in their ability to execute practices necessary to achieve specific outcomes, is a key factor in adopting and maintaining healthy practices, including diet, physical activity, and weight management (19, 20). The importance of self-efficacy in influencing health practices, particularly during adolescence and young adulthood, is well documented (18). Studies have shown that high self-efficacy enhances adherence to healthy dietary patterns, physical activity, and weight management behaviors, and is strongly associated with improved quality of life and better health indicators, consistent with previous research (3). Our findings reaffirm that stronger self-efficacy correlates with healthier lifestyle choices, underscoring the need to foster these beliefs early in life.

### 4.2 Cultural influence on self-efficacy

The findings also revealed that cultural context is a powerful determinant of self-efficacy across Arab regions, with significant regional differences in lifestyle-related self-efficacy. Young adults in northern Africa showed lower self-efficacy. This may be due to limited access to structured health education, economic disparities, and environmental constraints. These findings align with prior evidence that sociocultural norms, family expectations, and perceived barriers, such as time limitations and lack of motivation, can diminish confidence in adopting healthy behaviors (6, 21–23). In addition, self-efficacy (in all domains except exercise) was higher in both the Arabian Peninsula and Eastern Mediterranean regions. These results highlight the significant influence of the cultural context on self-efficacy beliefs, which is consistent with previous research showing that culture shapes the formation and expression of self-efficacy (21–26). Familial support plays a crucial role in reinforcing self-efficacy beliefs (5).

Self-efficacy in practicing exercise was particularly high in the Eastern Mediterranean, potentially due to community-based sports and social engagement opportunities, while lower scores in the Arabian Peninsula may be related to environmental barriers such as extreme heat (27–29). These regional variations highlight how context, opportunity, and support systems influence the confidence of an individual in engaging in health-promoting practices.

### 4.3 Knowledge and education

Nutritional knowledge was significantly higher in the Arabian Peninsula than in Northern Africa, followed by the Eastern Mediterranean region, indicating widespread access to health education and awareness campaigns. Nutrition education and awareness initiatives are instrumental in improving nutrition knowledge at school, university, and community levels throughout the Arabian Peninsula and have likely contributed to this outcome (30–32). The strong association between knowledge and self-efficacy aligns with existing literature, thereby emphasizing the role of education in empowering individuals to make informed health decisions (2, 5). These findings support Bandura’s social cognitive theory, which posits that knowledge enhances self-efficacy by providing individuals with the confidence to engage in health-promoting practices. This emphasizes the importance of strengthening knowledge regarding healthy lifestyles as a key strategy for boosting self-efficacy across all regions. Educational campaigns providing clear and accessible information on diet, exercise, and weight management may be particularly effective in empowering young adults to make healthier choices (2). Empowering young adults with practical and culturally relevant nutritional and exercise education can strengthen their confidence to adopt healthy habits. Public health policies should prioritize interactive, skill-based learning approaches such as digital health platforms, workshops, and gamified interventions to bridge knowledge gaps and simultaneously build self-efficacy.

### 4.4 Sociodemographic factors associated with self-efficacy

This study identified several key factors associated with self-efficacy in changing lifestyle practices among young Arab adults. Age and sex were significant factors associated with self-efficacy in the Eastern Mediterranean region and North Africa, consistent with prior literature linking sex and maturity to healthy behaviors. Self-efficacy is interrelated with cultural, environmental, physiological, and psychological factors (4, 33, 34). Efthymiou et al. reported that several sociodemographic factors are associated with self-efficacy, including parental age, educational level, and family income indices (2). Age, female sex, and nutritional knowledge were associated with high self-efficacy scores. Previous studies showed that women have higher dietary self-efficacy than men (2, 35). This finding aligns with existing literature, revealing that women, especially in traditional societies, may be more engaged in health-promoting practices. Greater responsibility is placed on women in numerous Arab cultures to manage their household health, including dietary choices, which may enhance their confidence in making lifestyle changes (36). These sex-influenced roles highlight the importance of designing equitable interventions to empower young men and women to engage in sustained lifestyle changes. Although age, sex, and nutritional knowledge were significantly associated with self-efficacy, the low R^2^ values in some regions (0.01–0.013) indicate that these factors explain only a small part of the variation. This is expected for complex behaviors like self-efficacy, which are shaped by many personal, social, and environmental influences (37).

### 4.5 Public health implications

The findings of the current study have important implications for the design of culturally sensitive health promotion strategies aimed at improving lifestyle practices across Arab regions. Significant regional disparities suggest that interventions should be tailored to address local realities, considering the cultural and sociocultural factors influencing self-efficacy in each region. For instance, programs in Northern Africa may need to focus on providing information and motivation to overcome barriers to healthy eating and physical activity (6), while health authorities could implement school- and community-based campaigns to enhance practical skills and knowledge. Interventions in the Eastern Mediterranean region should leverage existing social support networks to enhance self-efficacy (5) and policymakers can formalize community sports and social engagement programs to sustain healthy habits. In the Arabian Peninsula, where knowledge is relatively high, interventions should focus on translating awareness into action through structured exercise programs and hands-on nutrition workshops.

Future research should explore these relationships using longitudinal designs to better understand the causal pathways between culture, knowledge, sociodemographic factors, and self-efficacy. Qualitative studies may provide deeper insights into the cultural and social factors that influence self-efficacy in different Arab regions, thereby facilitating the development of more effective and culturally sensitive interventions.

The observed regional variations reinforce the need for contextually tailored health-promotion programs. Structured school-based health education can help address low confidence in nutrition and stress management, particularly where service access is uneven (38). National and regional policymakers should prioritize integrating interactive and skill-based health education into curricula and community programs. Digital and gamified interventions may enhance engagement and autonomy among youth, especially when sociocultural norms limit direct access to health resources (39). Community-based and family-inclusive programs are crucial in settings affected by displacement or health system strain, providing psychosocial support and practical health guidance (17). Across all contexts, training and empowering educators and counselors to reinforce health practices can further strengthen the self-efficacy of young Arab adults. Overall, policymakers and public health authorities should consider multi-level, culturally adapted strategies that address both individual and structural determinants of self-efficacy.

### 4.6 Limitations and future research

This study provides valuable insights into self-efficacy among young Arab adults, but several limitations should be noted. The cross-sectional design prevents establishing causal relationships, as data were collected at a single time point. The use of a convenience sampling approach and online data collection via social media may have introduced selection bias, over-representing educated, urban, and digitally connected participants, and under-representing those from rural or lower socioeconomic backgrounds. These factors limit generalizability to the broader young adult population across the Arab region.

Reliance on self-reported data may have introduced measurement bias, and moderate Cronbach’s alpha values may have attenuated associations and reduced reliability. A gender imbalance, particularly in the Arabian Peninsula sample, may have influenced regional estimates, as women often report higher self-efficacy (35). Future studies should consider region-specific analyses stratified by sex and use probability-based or mixed-method sampling to enhance representativeness.

Relatively low R^2^ values in several regression models suggest that additional factors, such as social support, income, environmental constraints, and other psychosocial influences, were not captured. Furthermore, although grouping countries into the Arabian Peninsula, Eastern Mediterranean, and North Africa facilitated broad cross-cultural comparisons, each region is internally heterogeneous in terms of political, economic, and demographic characteristics, which limits the validity of direct comparisons.

Despite these limitations, the observed cross-regional patterns provide preliminary evidence to guide culturally sensitive public health strategies and inform more methodologically robust future research on self-efficacy and lifestyle practices in Arab young adults.

## 5 Conclusion

Self-efficacy was lower in Northern African countries than in the Arabian Peninsula and Eastern Mediterranean countries, and age, sex, and nutritional knowledge were associated with higher self-efficacy. These findings reveal the importance of individual and sociodemographic factors on lifestyle practices among young Arab adults. Overall, the results underscore the need for culturally aware, demographically specific public health initiatives to strengthen self-efficacy and promote sustainable healthy behaviors across Arab countries.

## Data Availability

The raw data supporting the conclusions of this article will be made available by the authors, without undue reservation.
